# A qualitative quantitative mixed methods study of domestic violence against women

**DOI:** 10.1186/s12905-023-02483-0

**Published:** 2023-06-20

**Authors:** Mina Shayestefar, Mohadese Saffari, Razieh Gholamhosseinzadeh, Monir Nobahar, Majid Mirmohammadkhani, Seyed Hossein Shahcheragh, Zahra Khosravi

**Affiliations:** 1 School of Allied Medical Sciences, Semnan University of Medical Sciences, Semnan, Iran; 2 Amir Al Momenin Hospital, Social Security Organization, Ahvaz, Iran; 3 Department of Nursing, Faculty of Nursing and Midwifery, Semnan University of Medical Sciences, Semnan, Iran; 4 Social Determinants of Health Research Center, Semnan University of Medical Sciences, Semnan, Iran; 5 Clinical Research Development Unit, Kowsar Educational, Research and Therapeutic Hospital, Semnan University of Medical Sciences, Semnan, Iran; 6 Student Research Committee, School of Allied Medical Sciences, Semnan University of Medical Sciences, Semnan, Iran

**Keywords:** Domestic violence, Cross-sectional studies, Qualitative research, Iran

## Abstract

**Background:**

Violence against women is one of the most widespread, persistent and detrimental violations of human rights in today’s world, which has not been reported in most cases due to impunity, silence, stigma and shame, even in the age of social communication. Domestic violence against women harms individuals, families, and society. The objective of this study was to investigate the prevalence and experiences of domestic violence against women in Semnan.

**Methods:**

This study was conducted as mixed research (cross-sectional descriptive and phenomenological qualitative methods) to investigate domestic violence against women, and some related factors (quantitative) and experiences of such violence (qualitative) simultaneously in Semnan. In quantitative study, cluster sampling was conducted based on the areas covered by health centers from married women living in Semnan since March 2021 to March 2022 using Domestic Violence Questionnaire. Then, the obtained data were analyzed by descriptive and inferential statistics. In qualitative study by phenomenological approach and purposive sampling until data saturation, 9 women were selected who had referred to the counseling units of Semnan health centers due to domestic violence, since March 2021 to March 2022 and in-depth and semi-structured interviews were conducted. The conducted interviews were analyzed using Colaizzi’s 7-step method.

**Results:**

In qualitative study, seven themes were found including “Facilitators”, “Role failure”, “Repressors”, “Efforts to preserve the family”, “Inappropriate solving of family conflicts”, “Consequences”, and “Inefficient supportive systems”. In quantitative study, the variables of age, age difference and number of years of marriage had a positive and significant relationship, and the variable of the number of children had a negative and significant relationship with the total score and all fields of the questionnaire (p < 0.05). Also, increasing the level of female education and income both independently showed a significant relationship with increasing the score of violence.

**Conclusions:**

Some of the variables of violence against women are known and the need for prevention and plans to take action before their occurrence is well felt. Also, supportive mechanisms with objective and taboo-breaking results should be implemented to minimize harm to women, and their children and families seriously.

## Background

Violence against women by husbands (physical, sexual and psychological violence) is one of the basic problems of public health and violation of women’s human rights. It is estimated that 35% of women and almost one out of every three women aged 15–49 experience physical or sexual violence by their spouse or non-spouse sexual violence in their lifetime [1]. This is a nationwide public health issue, and nearly every healthcare worker will encounter a patient who has suffered from some type of domestic or family violence. Unfortunately, different forms of family violence are often interconnected. The “cycle of abuse” frequently persists from children who witness it to their adult relationships, and ultimately to the care of the elderly [2]. This violence includes a range of physical, sexual and psychological actions, control, threats, aggression, abuse, and rape [3].

Violence against women is one of the most widespread, persistent, and detrimental violations of human rights in today’s world, which has not been reported in most cases due to impunity, silence, stigma and shame, even in the age of social communication [3]. In the United States of America, more than one in three women (35.6%) experience rape, physical violence, and intimate partner violence (IPV) during their lifetime. Compared to men, women are nearly twice as likely (13.8% vs. 24.3%) to experience severe physical violence such as choking, burns, and threats with knives or guns [4]. The higher prevalence of violence against women can be due to the situational deprivation of women in patriarchal societies [5]. The prevalence of domestic violence in Iran reported 22.9%. The maximum of prevalence estimated in Tehran and Zahedan, respectively [6]. Currently, Iran has high levels of violence against women, and the provinces with the highest rates of unemployment and poverty also have the highest levels of violence against women [7].

Domestic violence against women harms individuals, families, and society [8]. Violence against women leads to physical, sexual, psychological harm or suffering, including threats, coercion and arbitrary deprivation of their freedom in public and private life. Also, such violence is associated with harmful effects on women’s sexual reproductive health, including sexually transmitted infection such as Human Immunodeficiency Virus (HIV), abortion, unsafe childbirth, and risky sexual behaviors [9]. There are high levels of psychological, sexual and physical domestic abuse among pregnant women [10]. Also, women with postpartum depression are significantly more likely to experience domestic violence during pregnancy [11].

Prompt attention to women’s health and rights at all levels is necessary, which reduces this problem and its risk factors [12]. Because women prefer to remain silent about domestic violence and there is a need to introduce immediate prevention programs to end domestic violence [13]. violence against women, which is an important public health problem, and concerns about human rights require careful study and the application of appropriate policies [14]. Also, the efforts to change the circumstances in which women face domestic violence remain significantly insufficient [15]. Given that few clear studies on violence against women and at the same time interviews with these people regarding their life experiences are available, the authors attempted to planning this research aims to investigate the prevalence and experiences of domestic violence against women in Semnan with the research question of “What is the prevalence of domestic violence against women in Semnan, and what are their experiences of such violence?”, so that their results can be used in part of the future planning in the health system of the society.

## Methods

This study is a combination of cross-sectional and phenomenology studies in order to investigate the amount of domestic violence against women and some related factors (quantitative) and their experience of this violence (qualitative) simultaneously in the Semnan city. This study has been approved by the ethics committee of Semnan University of Medical Sciences with ethic code of IR.SEMUMS.REC.1397.182. The researcher introduced herself to the research participants, explained the purpose of the study, and then obtained informed written consent. It was assured to the research units that the collected information will be anonymous and kept confidential. The participants were informed that participation in the study was entirely voluntary, so they can withdraw from the study at any time with confidence. The participants were notified that more than one interview session may be necessary. To increase the trustworthiness of the study, Guba and Lincoln’s criteria for rigor, including credibility, transferability, dependability, and confirmability [16], were applied throughout the research process. The COREQ checklist was used to assess the present study quality. The researchers used observational notes for reflexivity and it preserved in all phases of this qualitative research process.

### Qualitative method

Based on the phenomenological approach and with the purposeful sampling method, nine women who had referred to the counseling units of healthcare centers in Semnan city due to domestic violence in February 2021 to March 2022 were participated in the present study. The inclusion criteria for the study included marriage, a history of visiting a health center consultant due to domestic violence, and consent to participate in the study and unwillingness to participate in the study was the exclusion criteria. Each participant invited to the study by a telephone conversation about study aims and researcher information. The interviews place selected through agreement of the participant and the researcher and a place with the least environmental disturbance. Before starting each interview, the informed consent and all of the ethical considerations, including the purpose of the research, voluntary participation, confidentiality of the information were completely explained and they were asked to sign the written consent form. The participants were interviewed by depth, semi-structured and face-to-face interviews based on the main research question. Interviews were conducted by a female health services researcher with a background in nursing (M.Sh.). Data collection was continued until the data saturation and no new data appeared. Only the participants and the researcher were present during the interviews. All interviews were recorded by a MP3 Player by permission of the participants before starting. Interviews were not repeated. No additional field notes were taken during or after the interview.

The age range of the participants was from 38 to 55 years and their average age was 40 years. The sociodemographic characteristics of the participants are summarized in table below (Table 1).

**Table 1:** Sociodemographic characteristics of the participants

Participant	Age (years)	Educational level	Occupation	Husband’s age (year)	Husband’s educational level	Husband’s occupation
1	38	Literacy	Housewife	70	Literacy	Laborer
2	33	High school	Housewife	40	Diploma	Welder
3	32	Primary school	Housewife	35	Literacy	Laborer
4	54	Primary school	Housewife	59	Literacy	Drug dealer
5	39	Bachelor	Housewife	44	Bachelor	Office Worker
6	48	Diploma	Sewing	54	Diploma	Mostly unemployed
7	55	Literacy	Housewife	62	Diploma	Mostly unemployed with several dismissals from work
8	25	Literacy	Housewife	40	Literacy	Truck driver
9	36	Bachelor	Housewife	47	Diploma	Office Worker

Five interviews in the courtyards of healthcare centers, 2 interviews in the park, and 2 interviews at the participants’ homes were conducted. The duration of the interviews varied from 45 min to one hour. The main research question was “What is your experience about domestic violence?“. According to the research progress some other questions were asked in line with the main question of the research.

The conducted interviews were analyzed by using the 7 steps Colizzi’s method [17]. In order to empathize with the participants, each interview was read several times and transcribed. Then two researchers (M.Sh. and M.N.) extracted the phrases that were directly related to the phenomenon of domestic violence against women independently and distinguished from other sentences by underlining them. Then these codes were organized into thematic clusters and the formulated concepts were sorted into specific thematic categories.

In the final stage, in order to make the data reliable, the researcher again referred to 2 participants and checked their agreement with their perceptions of the content. Also, possible important contents were discussed and clarified, and in this way, agreement and approval of the samples was obtained.

### Quantitative method

The cross-sectional study was implemented from February 2021 to March 2022 with cluster sampling of married women in areas of 3 healthcare centers in Semnan city. Those participants who were married and agreed with the written and verbal informed consent about the ethical considerations were included to the study. The questionnaire was completed by the participants in paper and online form.

The instrument was the standard questionnaire of domestic violence against women by Mohseni Tabrizi et al. [18]. In the questionnaire, questions 1–10, 11–36, 37–65 and 66–71 related to sociodemographic information, types of spousal abuse (psychological, economical, physical and sexual violence), patriarchal beliefs and traditions and family upbringing and learning violence, respectively. In total, this questionnaire has 71 items.

The scoring of the questionnaire has two parts and the answers to them are based on the Likert scale. Questions 11–36 and 66–71 are answered with always [4] to never (0) and questions 37–65 with completely agree [4] to completely disagree (0). The minimum and maximum score is 0 and 300, respectively. The total score of 0–60, 61–120 and higher than 121 demonstrates low, moderate and severe domestic violence against women, respectively [18].

In the study by Tabrizi et al., to evaluate the validity and reliability of this questionnaire, researchers tried to measure the face validity of the scale by the previous research. Those items and questions which their accuracies were confirmed by social science professors and experts used in the research, finally. The total Cronbach’s alpha coefficient was 0.183, which confirmed that the reliability of the questions and items of the questionnaire is sufficient [18].

Descriptive data were reported using mean, standard deviation, frequency and percentage. Then, to measure the relationship between the variables, χ2 and Pearson tests also variance and regression analysis were performed. All analysis were performed by using SPSS version 26 and the significance level was considered as p < 0.05.

## Results

### Qualitative results

According to the third step of Colaizzi’s 7-step method, the researcher attempted to conceptualize and formulate the extracted meanings. In this step, the primary codes were extracted from the important sentences related to the phenomenon of violence against women, which were marked by underlining, which are shown below as examples of this stage and coding.

The primary code of indifference to the father’s role was extracted from the following sentences. This is indifference in the role of the father in front of the children.

“Some time ago, I told him that our daughter is single-sided deaf. She has a doctor’s appointment; I have to take her to the doctor. He said that I don’t have money to give you. He doesn’t force himself to make money anyway” (p 2, 33 yrs).

“He didn’t value his own children. He didn’t think about his older children” (p 4, 54 yrs).

The primary code extracted here included lack of commitment in the role of head of the household. This is irresponsibility towards the family and meeting their needs.

“My husband was fired from work after 10 years due to disorder and laziness. Since then, he has not found a suitable job. Every time he went to work, he was fired after a month because of laziness” (p 7, 55 yrs).

“In the evening, he used to get dressed and go out, and he didn’t come back until late. Some nights, I was so afraid of being alone that I put a knife under my pillow when I slept” (p 2, 33 yrs).

A total of 246 primary codes were extracted from the interviews in the third step. In the fourth step, the researchers put the formulated concepts (primary codes) into 85 specific sub-categories.

Twenty-three categories were extracted from 85 sub-categories. In the sixth step, the concepts of the fifth step were integrated and formed seven themes (Table 2).

**Table 2:** Extracted themes and categories from data analysis

Themes	Categories
Facilitators	***Husband’s criminal record*** ***Inappropriate age for marriage*** ***Selecting the wrong model for marriage*** ***Overdependence*** ***Bitter memories*** ***Ignorance***
Role failure	***Lack of commitment to different roles*** ***Resistance***
Repressors	***Fear and silence*** ***Normalcy*** ***Shame from society***
Efforts to preserve the family	***Hope and trust*** ***Efforts for children***
Inappropriate solving of family conflicts	***Child-bearing thoughts*** ***Lack of effective communication***
Consequences	***Harm to children*** ***After divorce*** ***Social harm*** ***Non-acceptance in the family*** ***Emotional harm***
Inefficient supportive systems	***Inappropriate family support*** ***Inefficiency of social systems*** ***Undesired training and advice***

**Fig. 1:**
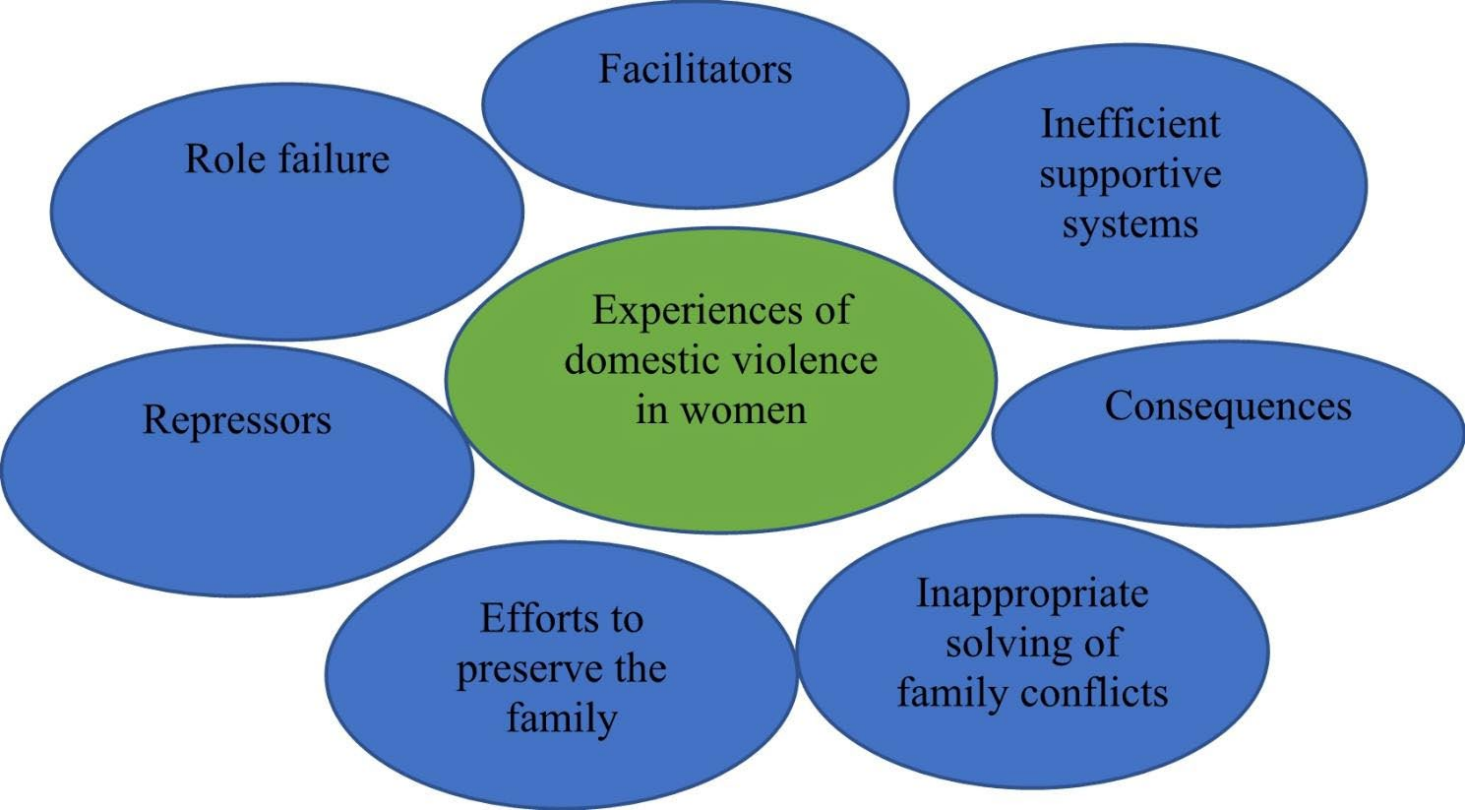
Themes of domestic violence against women

These themes included “Facilitators”, “Role failure”, “Repressors”, “Efforts to preserve the family”, “Inappropriate solving of family conflicts”, “Consequences”, and “Inefficient supportive systems” (Fig. 1).

Some of the statements of the participants on the theme of “**Facilitators”** are listed below:

#### Husband’s criminal record

“He got his death sentence for drugs. But, at last it was ended for 10 years” (p 4, 54 yrs).

#### Inappropriate age for marriage

“At the age of thirteen, I married a boy who was 25 years old” (p 8, 25 yrs).

“My first husband obeyed her parents. I was 12–13 years old” (p 3, 32 yrs).

“I couldn’t do anything. I was humiliated” (p 1, 38 yrs).

“A bridegroom came. The mother was against. She said, I am young. My older sister is not married yet, but I was eager to get married. I don’t know, maybe my father’s house was boring for me” (p 2, 33 yrs).

“My parents used to argue badly. They blamed each other and I always wanted to run away from these arguments. I didn’t have the patience to talk to mom or dad and calm them down” (p 5, 39 yrs).

#### Overdependence

“My husband’s parents don’t stop interfering, but my husband doesn’t say anything because he is a student of his father. My husband is self-employed and works with his father on a truck” (p 8, 25 yrs).

“Every time I argue with my husband because of lack of money, my mother-in-law supported her son and brought him up very spoiled and lazy” (p 7, 55 yrs).

#### Bitter memories

“After three years, my mother married her friend with my uncle’s insistence and went to Shiraz. But, his condition was that she did not have the right to bring his daughter with her. In fact, my mother also got married out of necessity” (p 8, 25 yrs).

Some of their other statements related to “**Role failure”** are mentioned below:

#### Lack of commitment to different roles

“I got angry several times and went to my father’s house because of my husband’s bad financial status and the fact that he doesn’t feel responsible to work and always says that he cannot find a job” (p 6, 48 yrs).

#### Resistance

“I saw that he does not want to change in any way” (p 4, 54 yrs).

“No matter how kind I am, it does not work” (p 1, 38 yrs).

Some of their other statements regarding “**Repressors”** are listed below:

#### Fear and silence

“My mother always forced me to continue living with my husband. Finally, my father had been poor. She all said that you didn’t listen to me when you wanted to get married, so you don’t have the right to get angry and come to me, I’m miserable enough” (p 2, 33 yrs).

“Because I suffered a lot in my first marital life. I was very humiliated. I said I would be fine with that. To be kind” (p1, 38 yrs).

#### Normalcy

“Well, I tell myself that he gets angry sometimes” (p 3, 32 yrs).

#### Shame from society

“I don’t want my daughter-in-law to know. She is not a relative” (p 4, 54 yrs).

Some of the statements of the participants regarding the theme of “**Efforts to preserve the family”** are listed below:

#### Hope and trust

“I always hope in God and I am patient” (p 2, 33 yrs).

#### Efforts for children

“My divorce took a month. We got a divorce. I forgave my dowry and took my children instead” (p 2, 33 yrs).

Some of their other statements regarding the “**Inappropriate solving of family conflicts”** are listed below:

#### Child-bearing thoughts

“My husband wanted to take me to a doctor to treat me. But my father-in-law refused and said that instead of doing this and spending money, marry again. Marriage in the clans was much easier than any other work” (p 8, 25 yrs).

#### Lack of effective communication

“I was nervous about him, but I didn’t say anything” (p 5, 39 yrs).

“Now I am satisfied with my life and thank God it is better to listen to people’s words. Now there is someone above me so that people don’t talk behind me” (p 2, 33 yrs).

Some of their other statements regarding the “**Consequences”** are listed below:

#### Harm to children

“My eldest daughter, who was about 7–8 years old, behaved differently. Oh, I was angry. My children are mentally depressed and argue” (p 5, 39 yrs).

#### After divorce

“Even though I got a divorce, my mother and I came to a remote area due to the fear of what my family would say” (p 2, 33 yrs).

#### Social harm

“I work at a retirement center for living expenses” (p 2, 33 yrs).

“I had to go to clean the houses” (p 5, 39 yrs).

#### Non-acceptance in the family

“The children’s relationship with their father became bad. Because every time they saw their father sitting at home smoking, they got angry” (p 7, 55 yrs).

#### Emotional harm

“When I look back, I regret why I was not careful in my choice” (p 7, 55 yrs).

“I felt very bad. For being married to a man who is not bound by the family and is capricious” (p 9, 36 yrs).

Some of their other statements regarding “**Inefficient supportive systems”** are listed below:

#### Inappropriate family support

“We didn’t have children. I was at my father’s house for about a month. After a month, when I came home, I saw that my husband had married again. I cried a lot that day. He said, God, I had to. I love you. My heart is broken, I have no one to share my words” (p 8, 25 yrs).

“My brother-in-law was like himself. His parents had also died. His sister did not listen at all” (p 4, 54 yrs).

“I didn’t have anyone and I was alone” (p 1, 38 yrs).

#### Inefficiency of social systems

“That day he argued with me, picked me up and threw me down some stairs in the middle of the yard. He came closer, sat on my stomach, grabbed my neck with both of his hands and wanted to strangle me. Until a long time later, I had kidney problems and my neck was bruised by her hand. Given that my aunt and her family were with us in a building, but she had no desire to testify and was afraid” (p 3, 32 yrs).

#### Undesired training and advice

“I told my mother, you just said no, how old I was? You never insisted on me and you didn’t listen to me that this man is not good for you” (p 9, 36 yrs).

### Quantitative results

In the present study, 376 married women living in Semnan city participated in this study. The mean age of participants was 38.52 ± 10.38 years. The youngest participant was 18 and the oldest was 73 years old. The maximum age difference was 16 years. The years of marriage varied from one year to 40 years. Also, the number of children varied from no children to 7. The majority of them had 2 children (109, 29%). The sociodemographic characteristics of the participants are summarized in the table below (Table 3).

**Table 3:** Sociodemographic characteristics

Variable		Study sample (n = 376)
Age (year)		38.52 ± 10.38^a^
Age difference with husband (year)		3.25 ± 2.96 ^a^
Years of marriage		8.20 ± 8.26 ^a^
No. of children		2.54 ± 1.29 ^a^
Educational level	Primary or high school	199 (52.8)^b^
	University	177 (47.1) ^b^
Husband’s educational level	Primary or high school	216 (57.4) ^b^
	University	160 (42.6) ^b^
Occupation	Housewife	237 (63) ^b^
	Office Worker	107 (28.5) ^b^
	Manual worker	32 (8.5) ^b^
Husband’s occupation	Unemployed	10 (2.7) ^b^
	Office Worker	120 (31.9) ^b^
	Manual worker	147 (39.1) ^b^

^a^ Mean ± SD

^b^ Frequencies (%)

The frequency distribution (number and percentage) of the participants in terms of the level of violence was as follows. 89 participants (23.7%) had experienced low violence, 59 participants (15.7%) had experienced moderate violence, and 228 participants (60.6%) had experienced severe violence.

Cronbach’s alpha for the reliability of the questionnaire was 0.988. The mean and standard deviation of the total score of the questionnaire was 143.60 ± 74.70 with a range of 3-244. The relationship between the total score of the questionnaire and its fields, and some demographic variables is summarized in the table below (Table 4).

**Table 4:** Correlation between the demographic variables with total and each domain scores of the questionnaire

Variable	Mean ± SD	Age		Age difference (year)		Years of marriage		Number of children		Difference of Education level	
		r	p-value	r	p-value	r	p-value	r	p-value	r	p-value
Psychological violence	24.99 ± 17.18	0.24	< 0.001	0.44	< 0.001	0.36	< 0.001	-0.13	0.007	0.03	0.48
Economical violence	11.56 ± 8.62	0.23	< 0.001	0.44	< 0.001	0.37	< 0.001	-0.17	0.001	0.04	0.39
Physical violence	16.78 ± 12.89	0.25	< 0.001	0.47	< 0.001	0.42	< 0.001	-0.19	< 0.001	0.05	0.32
Sexual violence	7 ± 5.25	0.25	< 0.001	0.43	< 0.001	0.35	< 0.001	-0.15	0.003	0.01	0.82
**Domestic violence**	60.35 ± 42.96	0.25	0.002	0.36	< 0.001	0.39	< 0.001	-0.16	0.001	0.04	0.43
Patriarchy beliefs	69.60 ± 29.98	0.16	< 0.001	0.32	< 0.001	0.30	< 0.001	-0.18	< 0.001	0.03	0.56
Tradition and family upbringing	13.64 ± 8.82	0.26	< 0.001	0.43	< 0.001	0.31	< 0.001	-0.13	0.01	-0.003	0.94
**Total score**	143.60 ± 74.70	0.24	< 0.001	0.44	< 0.001	0.38	< 0.001	-0.18	< 0.001	0.03	0.5

As shown in the table above, the variables of age, age difference and number of years of marriage have a positive and significant relationship, and the variable of number of children has a negative and significant relationship with the total score and all fields of the questionnaire (p < 0.05). However, the variable of education level difference showed no significant relationship with the total score and any of the fields. Also, the highest average score is related to patriarchal beliefs compared to other fields.

The comparison of the average total scores separately according to each variable showed the significant average difference in the variables of the previous marriage history of the woman, the result of the previous marriage of the woman, the education of the woman, the education of the man, the income of the woman, the income of the man, and the physical disease of the man (p < 0.05).

In the regression model, two variables remained in the final model, indicating the relationship between the variables and violence score and the importance of these two variables. An increase in women’s education and income level both independently show a significant relationship with an increase in violence score (Table 5).

**Table 5:** Regression analysis of predicting violence

Coefficients					
Variable	Unstandardized Coefficients		Standardized Coefficients	t	p-value
	B	SE	β		
Women’s education	3.386	1.015	0.237	3.334	0.001
Women’s income	3.439	1.038	0.235	3.312	0.001

The results of analysis of variance to compare the scores of each field of violence in the subgroups of the participants also showed that the experience and result of the woman’s previous marriage has a significant relationship with physical violence and tradition and family upbringing, the experience of the man’s previous marriage has a significant relationship with patriarchal belief, the education level of the woman has a significant relationship with all fields and the level of education of the man has a significant relationship with all fields except tradition and family upbringing (p < 0.05).

## Discussion

According to the results of both quantitative and qualitative studies, variables such as the young age of the woman and a large age difference are very important factors leading to an increase in violence. At a younger age, girls are afraid of the stigma of society and family, and being forced to remain silent can lead to an increase in domestic violence. As Gandhi et al. (2021) stated in their study in the same field, a lower marriage age leads to many vulnerabilities in women. Early marriage is a global problem associated with a wide range of health and social consequences, including violence for adolescent girls and women [12]. Also, Ahmadi et al. (2017) found similar findings, reporting a significant association among IPV and women age ≤ 40 years [19].

Two others categories of “Facilitators” in the present study were “Husband’s criminal record” and “Overdependence” which had a sub-category of “Forced cohabitation”. Ahmadi et al. (2017) reported in their population-based study in Iran that husband’s addiction and rented-householders have a significant association with IPV [19].

The patriarchal beliefs, which are rooted in the tradition and culture of society and family upbringing, scored the highest in relation to domestic violence in this study. On the other hand, in qualitative study, “Normalcy” of men’s anger and harassment of women in society is one of the “Repressors” of women to express violence. In the quantitative study, the increase in the women’s education and income level were predictors of the increase in violence. Although domestic violence is more common in some sections of society, women with a wide range of ages, different levels of education, and at different levels of society face this problem, most of which are not reported. Bukuluki et al. (2021) showed that women who agreed that it is good for a man to control his partner were more likely to experience physical violence [20].

Domestic violence leads to “Consequences” such as “Harm to children”, “Emotional harm”, “Social harm” to women and even “Non-acceptance in their own family”. Because divorce is a taboo in Iranian culture and the fear of humiliating women forces them to remain silent against domestic violence. Balsarkar (2021) stated that the fear of violence can prevent women from continuing their studies, working or exercising their political rights [8]. Also, Walker-Descarte et al. (2021) recognized domestic violence as a type of child maltreatment, and these abusive behaviors are associated with mental and physical health consequences [21].

On the other hand and based on the “Lack of effective communication” category, ignoring the role of the counselor in solving family conflicts and challenges in the life of couples in the present study was expressed by women with reasons such as lack of knowledge and family resistance to counseling. Several pathologies are needed to investigate increased domestic violence in situations such as during women’s pregnancy or infertility. Because the use of counseling for couples as a suitable solution should be considered along with their life challenges. Lin et al. (2022) stated that pregnant women were exposed to domestic violence for low birth weight in full term delivery. Spouse violence screening in the perinatal health care system should be considered important, especially for women who have had full-term low birth weight infants [22].

Also, lack of knowledge and low level of education have been found as other factors of violence in this study, which is very prominent in both qualitative and quantitative studies. Because the social systems and information about the existing laws should be followed properly in society to act as a deterrent. Psychological training and especially anger control and resilience skills during education at a younger age for girls and boys should be included in educational materials to determine the positive results in society in the long term. Manouchehri et al. (2022) stated that it seems necessary to train men about the negative impact of domestic violence on the current and future status of the family [23]. Balsarkar (2021) also stated that men and women who have not had the opportunity to question gender roles, attitudes and beliefs cannot change such things. Women who are unaware of their rights cannot claim. Governments and organizations cannot adequately address these issues without access to standards, guidelines and tools [8]. Machado et al. (2021) also stated that gender socialization reinforces gender inequalities and affects the behavior of men and women. So, highlighting this problem in different fields, especially in primary health care services, is a way to prevent IPV against women [24].

There was a sub-category of “Inefficiency of social systems” in the participants experiences. Perhaps the reason for this is due to insufficient education and knowledge, or fear of seeking help. Holmes et al. (2022) suggested the importance of ascertaining strategies to improve victims’ experiences with the court, especially when victims’ requests are not met, to increase future engagement with the system [25]. Sigurdsson (2019) revealed that despite high prevalence numbers, IPV is still a hidden and underdiagnosed problem and neither general practitioner nor our communities are as well prepared as they should be [26]. Moreira and Pinto da Costa (2021) found that while victims of domestic violence often agree with mandatory reporting, various concerns are still expressed by both victims and healthcare professionals that require further attention and resolution [27]. It appears that legal and ethical issues in this regard require comprehensive evaluation from the perspectives of victims, their families, healthcare workers, and legal experts. By doing so, better practical solutions can be found to address domestic violence, leading to a downward trend in its occurrence.

## Conclusions

Some of the variables of violence against women have been identified and emphasized in many studies, highlighting the necessity of policymaking and social pathology in society to prevent and use operational plans to take action before their occurrence. Breaking the taboo of domestic violence and promoting divorce as a viable solution after counseling to receive objective results should be implemented seriously to minimize harm to women, children, and their families.

### Limitations

Domestic violence against women is an important issue in Iranian society that women resist showing and expressing, making researchers take a long-term process of sampling in both qualitative and quantitative studies. The location of the interview and the women’s fear of their husbands finding out about their participation in this study have been other challenges of the researchers, which, of course, they attempted to minimize by fully respecting ethical considerations. Despite the researchers’ efforts, their personal and professional experiences, as well as the studies reviewed in the literature review section, may have influenced the study results.

## Data Availability

Data and materials will be available upon email to the corresponding author.

## List of Abbreviation

IPV: Intimate Partner Violence
HIV: Human Immunodeficiency Virus

## References

[1] Organization WH. Violence against women prevalence estimates, 2018: global, regional and national prevalence estimates for intimate partner violence against women and global and regional prevalence estimates for non-partner sexual violence against women. World Health Organization; 2021.

[2] Huecker MR, Malik A, King KC, Smock W. Kentucky Domestic Violence. StatPearls. Treasure Island (FL) ineligible companies. Disclosure: Ahmad Malik declares no relevant financial relationships with ineligible companies. Disclosure: Kevin King declares no relevant financial relationships with ineligible companies. Disclosure: William Smock declares no relevant financial relationships with ineligible companies.: StatPearls Publishing Copyright © 2023, StatPearls Publishing LLC.; 2023.

[3] Gandhi A, Bhojani P, Balkawade N, Goswami S, Kotecha Munde B, Chugh A (2021). Analysis of survey on violence against women and early marriage: Gyneaecologists’ perspective. J Obstet Gynecol India.

[4] Sugg N (2015). Intimate partner violence: prevalence, health consequences, and intervention. Med Clin.

[5] Abebe Abate B, Admassu Wossen B, Tilahun Degfie T (2016). Determinants of intimate partner violence during pregnancy among married women in Abay Chomen district, western Ethiopia: a community based cross sectional study. BMC Womens Health.

[6] Adineh H, Almasi Z, Rad M, Zareban I, Moghaddam A (2016). Prevalence of domestic violence against women in Iran: a systematic review. Epidemiol (Sunnyvale).

[7] Pirnia B, Pirnia F, Pirnia K (2020). Honour killings and violence against women in Iran during the COVID-19 pandemic. The Lancet Psychiatry.

[8] Balsarkar G (2021). Summary of four recent studies on violence against women which obstetrician and gynaecologists should know. J Obstet Gynecol India.

[9] Ellsberg M, Jansen HA, Heise L, Watts CH, Garcia-Moreno C (2008). Intimate partner violence and women’s physical and mental health in the WHO multi-country study on women’s health and domestic violence: an observational study. The lancet.

[10] Chasweka R, Chimwaza A, Maluwa A (2018). Isn’t pregnancy supposed to be a joyful time? A cross-sectional study on the types of domestic violence women experience during pregnancy in Malawi. Malawi Med journal: J Med Association Malawi.

[11] Afshari P, Tadayon M, Abedi P, Yazdizadeh S (2020). Prevalence and related factors of postpartum depression among reproductive aged women in Ahvaz. Iran Health care women Int.

[12] Gebrezgi BH, Badi MB, Cherkose EA, Weldehaweria NB (2017). Factors associated with intimate partner physical violence among women attending antenatal care in Shire Endaselassie town, Tigray, northern Ethiopia: a cross-sectional study, July 2015. Reproductive health.

[13] Duran S, Eraslan ST (2019). Violence against women: affecting factors and coping methods for women. J Pak Med Assoc.

[14] Devries KM, Mak JY, Garcia-Moreno C, Petzold M, Child JC, Falder G (2013). The global prevalence of intimate partner violence against women. Science.

[15] Mahapatro M, Kumar A (2021). Domestic violence, women’s health, and the sustainable development goals: integrating global targets, India’s national policies, and local responses. J Public Health Policy.

[16] Lincoln YS, Guba EG. Naturalistic inquiry: sage; 1985.

[17] Colaizzi PF. Psychological research as the phenomenologist views it. 1978.

[18] Mohseni Tabrizi A, Kaldi A, Javadianzadeh M (2013). The study of domestic violence in Marrid Women Addmitted to Yazd Legal Medicine Organization and Welfare Organization. Tolooebehdasht.

[19] Ahmadi R, Soleimani R, Jalali MM, Yousefnezhad A, Roshandel Rad M, Eskandari A (2017). Association of intimate partner violence with sociodemographic factors in married women: a population-based study in Iran. Psychol Health Med.

[20] Bukuluki P, Kisaakye P, Wandiembe SP, Musuya T, Letiyo E, Bazira D (2021). An examination of physical violence against women and its justification in development settings in Uganda. PLoS ONE.

[21] Walker-Descartes I, Mineo M, Condado LV, Agrawal N (2021). Domestic violence and its Effects on Women, Children, and families. Pediatr Clin North Am.

[22] Lin C-H, Lin W-S, Chang H-Y, Wu S-I (2022). Domestic violence against pregnant women is a potential risk factor for low birthweight in full-term neonates: a population-based retrospective cohort study. PLoS ONE.

[23] Manouchehri E, Ghavami V, Larki M, Saeidi M, Latifnejad Roudsari R (2022). Domestic violence experienced by women with multiple sclerosis: a study from the North-East of Iran. BMC Womens Health.

[24] Machado DF, Castanheira ERL, Almeida MASd (2021). Intersections between gender socialization and violence against women by the intimate partner. Ciência & Saúde Coletiva.

[25] Holmes SC, Maxwell CD, Cattaneo LB, Bellucci BA, Sullivan TP (2022). Criminal Protection orders among women victims of intimate Partner violence: Women’s Experiences of Court decisions, processes, and their willingness to Engage with the system in the future. J interpers Violence.

[26] Sigurdsson EL (2019). Domestic violence-are we up to the task?. Scand J Prim Health Care.

[27] Moreira DN, Pinto da Costa M (2021). Should domestic violence be or not a public crime?. J Public Health.

